# Stimulation of ectopically expressed muscarinic receptors induces IFN-γ but suppresses IL-2 production by inhibiting activation of pAKT pathways in primary T cells

**DOI:** 10.1073/pnas.2300987120

**Published:** 2023-06-12

**Authors:** Trang T. T. Nguyen, Wen Lu, Wandi S. Zhu, K. Mark Ansel, Hong-Erh Liang, Arthur Weiss

**Affiliations:** ^a^Rosalind Russell-Ephraim Engleman Rheumatology Research Center, Division of Rheumatology, Department of Medicine, University of California, San Francisco, CA 94143; ^b^Department of Microbiology and Immunology, University of California San Francisco, San Francisco, CA 94143; ^c^Department of Medicine, University of California San Francisco, San Francisco, 94143

**Keywords:** muscarinic receptor, GPCR, T cells, signaling

## Abstract

T cell antigen receptor stimulation induces tyrosine phosphorylation of downstream signaling molecules and the phosphatidylinositol, Ras, MAPK, and PI3 kinase pathways, leading to T cell activation. Previously, we reported that the G-protein-coupled human muscarinic receptor could bypass tyrosine kinases to activate the phosphatidylinositol pathway and induce interleukin-2 production in Jurkat leukemic T cells. Here, we demonstrate that stimulating G-protein-coupled muscarinic receptors (M1 and synthetic hM3Dq) can activate primary mouse T cells if PLCβ1 is coexpressed. Resting peripheral hM3Dq+PLCβ1 (hM3Dq/β1) T cells did not respond to clozapine, an hM3Dq agonist, unless they were preactivated by TCR and CD28 stimulation which increased hM3Dq and PLCβ1 expression. This permitted large calcium and phosphorylated ERK responses to clozapine. Clozapine treatment induced high IFN-γ, CD69, and CD25 expression, but surprisingly did not induce substantial IL-2 in hM3Dq/β1 T cells. Importantly, costimulation of both muscarinic receptors plus the TCR even led to reduced IL-2 expression, suggesting a selective inhibitory effect of muscarinic receptor costimulation. Stimulation of muscarinic receptors induced strong nuclear translocation of NFAT and NFκB and activated AP-1. However, stimulation of hM3Dq led to reduced IL-2 mRNA stability which correlated with an effect on the IL-2 3′UTR activity. Interestingly, stimulation of hM3Dq resulted in reduced pAKT and its downstream pathway. This may explain the inhibitory impact on IL-2 production in hM3Dq/β1T cells. Moreover, an inhibitor of PI3K reduced IL-2 production in TCR-stimulated hM3Dq/β1 CD4 T cells, suggesting that activating the pAKT pathway is critical for IL-2 production in T cells.

## Significance

Studying the functions of G-protein-coupled muscarinic receptors, which are protein tyrosine kinase independent, might inspire new cancer therapies by bypassing classical TCR signaling pathways. Stimulating heterologously expressed muscarinic receptors (M1 and the synthetic hM3Dq) induced calcium responses and phosphorylation of ERK in preactivated T cells if PLCβ1 was coexpressed. Unexpectedly, whereas stimulation of hM3Dq that couples to PLCβ1 induced high IFN-γ, CD69, and CD25 expression, surprisingly, it did not induce high IL-2 expression. Stimulation of hM3Dq reduced IL-2 mRNA stability which correlated with an effect on the IL-2 mRNA stability. The selective effect on IL-2 mRNA may be attributable to reduced pAKT downstream pathway function, suggesting that the pAKT pathway is critical for IL-2 production.

TCR-induced downstream signaling events leading to T cell activation depend on protein tyrosine kinase (PTK)–dependent pathways (1, 2). Upon engagement of the TCR, the PTKs Lck and ZAP-70 participate in initiating TCR signaling events, leading to the phosphorylation of the adaptors LAT and SLP-76. Phosphorylation of tyrosine residues on LAT results in the recruitment of phospholipase Cγ1 (PLCγ1) and the other effector molecules, such as Itk which phosphorylates and activates PLCγ1 activity. This leads to the activation of the PKC and Ras pathways and induces calcium increases (3). These PTK-dependent activities are essential during TCR signaling (1). On the contrary, the immune system employs immunoreceptor tyrosine–based inhibition motifs (ITIMs) that recruit protein tyrosine phosphatases (PTPs), such as SHP-1 and SHP-2 to inhibit the PTK-dependent signaling pathways as well as the lipid phosphatases SHIP-1 and -2 (1, 2). T cell unresponsiveness in anergic and exhausted T cells has been attributed, in part, to the induction of multiple negative regulators that target T cell–dependent PTK-dependent pathways (4, 5). In Jurkat leukemic T cells, we previously found that G-protein-coupled muscarinic receptors can be used to activate and induce IL-2 production, thereby bypassing the requirement for PTK-dependent TCR signaling pathways (6–8). We considered the possibility that stimulating T cells via ectopically expressed muscarinic receptors, whose functions are PTK independent, might contribute to the design of unique therapeutic strategies for cancers by bypassing or enhancing classical TCR signaling pathways and T cell responses to this PTK-independent class of receptors.

Muscarinic receptors belong to the G-protein-coupled receptor (GPCR) family and are composed of five receptor subtypes (M1, M2, M3, M4, and M5) (9). These receptors are widely distributed in multiple organs and tissues and are critical for the maintenance of central and peripheral cholinergic neurotransmission. The five muscarinic receptor subtypes can be further divided into two major functional classes based on their specific G-protein-coupling properties. While the M1, M3, and M5 receptor subtypes selectively couple to the Gq proteins, the M2 and M4 receptor subtypes preferentially activate Gi and Go proteins (10). Stimulation of M1, M3, and M5 receptor subtypes activates Gq proteins, leading to activation of the membrane-bound phospholipase Cβ (PLCβ) enzymes (9). This results in increases in cytoplasmic free calcium concentrations and activation of PKC and the Ras/MAPK pathways (9, 10), signaling pathways critically important for the induction of numerous cytokines and other events associated with T cell activation. We have previously shown that the stimulation of the human M1 receptor (hM1) bypasses PTK-dependent TCR pathways to induce calcium increases, ERK activation, and IL-2 production in the human Jurkat T cell leukemic line (6–8).

To study the biochemical signaling and functional consequences of stimulating muscarinic receptors in primary T cells, we used two muscarinic receptors: 1) the murine M1 receptor, which is activated by the native ligand acetylcholine or by a pharmacologically agonist carbachol, and 2) an engineered human M3 receptor named Gq-coupled hM3 DREADD “hM3Dq” (DREADD: designer receptors exclusively activated by designer drugs), which can be selectively activated by the normally physiologically inert synthetic ligand clozapine (11, 12). Using this system, we found that high-level expression of PLCβ1 in T cells was required, as was high-level expression of the muscarinic receptors, for effective signaling. Stimulation of the muscarinic receptors induced many of the events associated with T cell activation via the TCR. However, we also identified an unanticipated requirement for AKT pathway activity for IL-2 production but not for several other cytokines, as well as an inhibitory effect of the muscarinic receptors on the AKT pathway.

## Results

### PLCβ1 Greatly Enhances Calcium Responses by Activated Muscarinic Receptors in Primary T Cells.

**Fig. 1.**
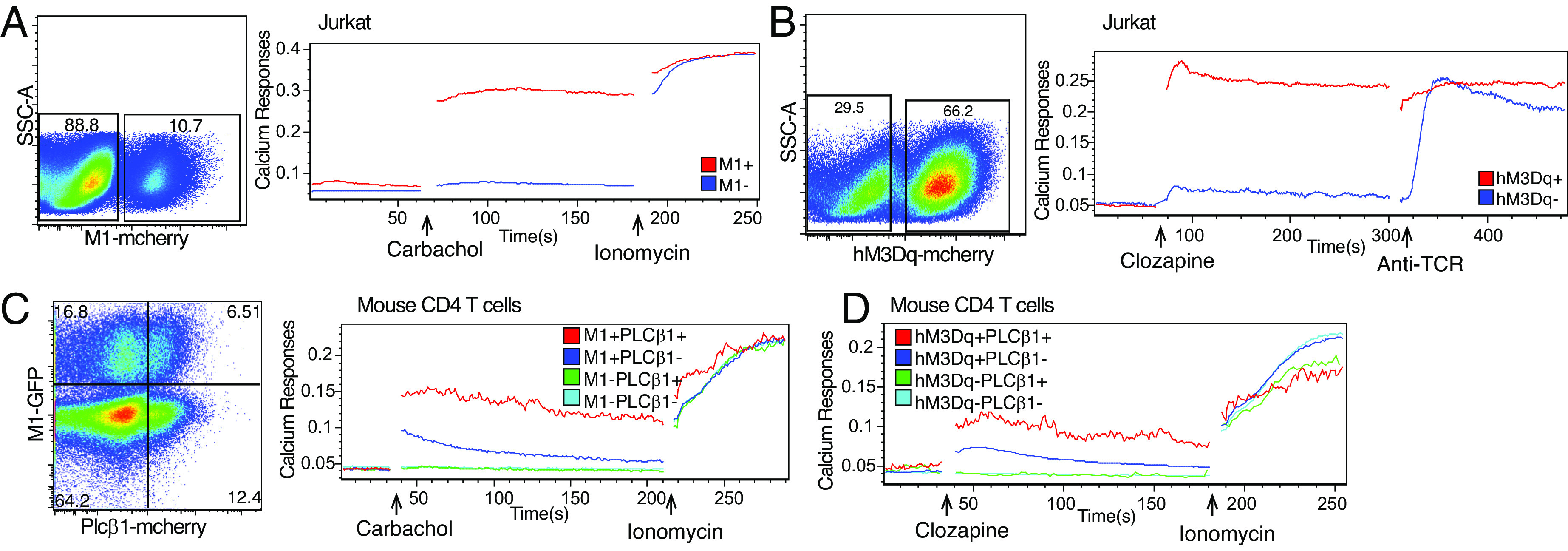
PLCβ1 greatly enhances calcium responses in activated muscarinic receptors in primary T cells. (*A* and *B*) Lentivirus transfer vectors encoding M1-mCherry or hM3Dq-mCherry were used to transduce muscarinic receptors into Jurkat cells. Cells transduced with M1 or hM3Dq were gated on mCherry-positive cells. Calcium changes in response to carbachol (M1 agonist, 500 μM) or clozapine (hM3Dq agonist, 10 μM) and ionomycin (1 μM) or anti-Jurkat TCR (C305, 1 μg/mL) over time. (*C* and *D*) Primary mouse CD4 T cells were transduced by retrovirus transfer vectors encoding M1-GFP or hM3Dq-GFP with PLCβ1-mCherry. Calcium changes in M1 or hM3Dq transduced primary CD4 T cells in response to carbachol (M1 agonist, 500 μM) or clozapine (hM3Dq agonist, 10 μM) and ionomycin (1 μM) over time.

We first studied the biochemical signaling and functions of two types of muscarinic receptors, M1 (flag epitope tagged) and hM3Dq (HA epitope tagged), which can be stimulated by carbachol or clozapine, respectively, in Jurkat leukemic T cells. Transduction of Jurkat cells with lentiviruses containing either muscarinic receptor (M1 or hM3Dq) led to results that were consistent with our previous findings (6, 7), namely, that stimulation of muscarinic receptors (M1 or hM3Dq) induced strong calcium responses in Jurkat cells (Fig. 1 *A* and *B*). Muscarinic receptors were then virally transduced into primary mouse T cells to determine whether their stimulation can induce signaling in these cells. Retroviral transduction of either muscarinic receptor (M1 or hM3Dq) in primary mouse T cells showed that both of these receptors were responsive to stimulation with carbachol or clozapine, respectively, at least with regard to calcium increases (Fig. 1 *C* and *D*). However, the magnitudes of these responses were much lower than those observed in Jurkat cells (Fig. 1 *A*–*D*). PLCβ, downstream of Gq, is activated by muscarinic receptor stimulation. The predominant isoforms expressed in Jurkat cells are the PLCβ1, 2, and 3 isoforms (13) (*SI Appendix*, Fig. S1*A*). Neuronal cells, which naturally express different types of muscarinic receptors (14), exhibited the highest expression of PLCβ1 among all PLCβ isoforms (*SI Appendix*, Fig. S1*B*). In contrast, mouse-naïve or activated CD4 and CD8 T cells did not express PLCβ1 either at the mRNA and protein levels (*SI Appendix*, Fig. S1 *C–F*). The predominant PLCβ isoforms expressed in primary human and mouse CD4 and CD8 T lymphocytes are PLCβ2 and PLCβ3 (13, 15) (*SI Appendix*, Fig. S1 *C–F*). Significantly, PLCβ1 is important in mediating optimal IL-2 production in response to superantigens in Jurkat cells (16). Therefore, we determined whether transduction of PLCβ1 in mouse T cells could improve responses to stimulated muscarinic receptors in mouse T cells. Fortunately, viral transduction of PLCβ1 strongly amplified the induced calcium responses of either of the stimulated muscarinic receptors (Fig. 1 *C* and *D*). Thus, the transduced PLCβ1 appeared to more efficiently mediate signals from the stimulated transduced muscarinic receptors than the endogenously expressed PLCβ isoforms.

### hM3Dq+ PLCβ1+ (hM3Dq/β1) T Cells Preactivated by TCR and CD28 Stimulation Exhibited Large Calcium and pErk Responses to hM3Dq Agonist.

**Fig. 2.**
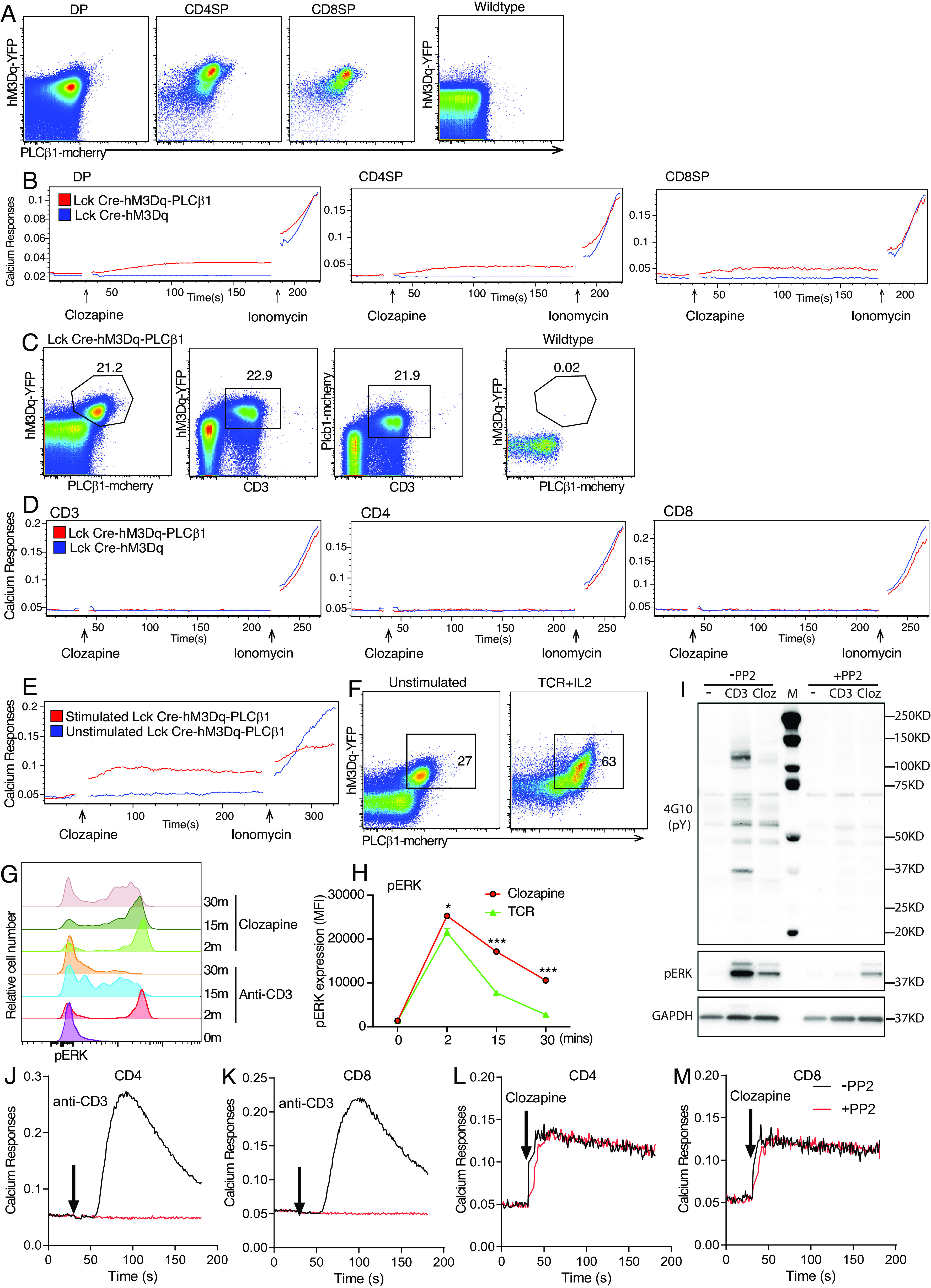
hM3Dq+ PLCβ1+ (hM3Dq/β1) T cells preactivated by TCR and CD28 stimulation exhibited large calcium and pErk responses to hM3Dq agonist. (*A*) hM3Dq-YFP and PLCβ1-mCherry expression in DP, CD4SP, and CD8SP thymocytes from Lck-Cre x hM3Dq x PLCβ1 and wild-type mice. (*B*) Calcium changes in Lck-Cre × hM3Dq × PLCβ1 thymocytes. (*C*) hM3Dq-YFP and PLCβ1-mCherry expression in peripheral T cells from LckCre-hM3Dq–PLCβ1 mice. (*D*) Calcium changes in peripheral Lck-Cre × hM3Dq × PLCβ1 T cells. (*E* and *F*) CD4 T cells from LckCre-hM3Dq–PLCβ1 mice were activated with anti-CD3 (1 μg/mL) + anti-CD28 (2 μg/mL) and IL-2 (40 U/mL) for 3 d and then cultured with IL-2 (40 U/mL) for 2 d. (*E*) Calcium changes in stimulated and unstimulated peripheral Lck-Cre × hM3Dq × PLCβ1 CD4 T cells. (*F*) hM3Dq-YFP and PLCβ1-mCherry expression in stimulated and unstimulated LckCre-hM3Dq–PLCβ1 CD4 T cells. (*G* and *H*) Overlaid histograms and bar chart showing mean fluorescent intensity (MFI) of phosphorylated Erk in hM3Dq/β1 CD4 T cells in response to 10 μM clozapine or 10 μg/mL anti-CD3 followed by cross-linking with 20 μg/mL anti-Armenian hamster IgG over time. (*I*) Immunoblot analysis of phosphotyrosine, phosphorylated Erk, Gapdh (loading control) in hM3Dq/β1 CD4 T cells pretreated with or without PP2 (40 μM) and then stimulated with 10 μM clozapine or 0.1 μg/mL anti-CD3 followed by cross-linking with 20 μg/mL anti-Armenian hamster IgG for 5 min. M, molecular-weight size markers. Data are representative of three independent experiments. (*J*–*M*) Calcium responses in hM3Dq/β1 CD4 and CD8 T cells pretreated with or without PP2 (40 μM) and then stimulated with 0.1 μg/mL anti-CD3 followed by cross-linking with 20 μg/mL anti-Armenian hamster IgG or 10 μM clozapine. *P < 0.05, **P < 0.005, ***P < 0.0005.

We generated mice with the properly inserted PLCβ1cDNA in the Rosa 26 locus (*SI Appendix*, Fig. S2 *A* and *B*). The expression of this PLCβ1 transgene in T cells was validated by protein blot analysis (*SI Appendix*, Fig. S2*C*) and could be monitored by the expression of the fluorescent protein mCherry separated from PLCβ1 by an IRES sequence (*SI Appendix*, Fig. S2 *A* and *B*). Inducible PLCβ1 knock-in mice were crossed with hM3Dq x Lck-Cre mice to generate mice in which only T cells express both hM3Dq and PLCβ1 to study the muscarinic receptor–regulated activation of PLCβ1 in T cells (*SI Appendix*, Fig. S2 *D* and *E*). Cre-inducible hM3Dq transgenic mice (17) have the transgene integrated on chromosome 14, which has not adversely affected the functionality of the allele (https://www.jax.org/strain/026220). Lck Cre–mediated removal of an upstream floxed-STOP cassette allows hM3Dq-mcitrine and PLCβ1-mcherry to be expressed in T cells in Lck-Cre-hM3Dq-PLCβ1 mice. All subsequent experiments used only Lck-Cre-hM3Dq–PLCβ1 T cells that expressed both hM3Dq and PLCβ1. For simplicity, we labeled these cells as hM3Dq/β1 T cells. Thymocytes showed increased expression of hM3Dq and PLCβ1 from CD4SP and CD8SP relative to the amounts expressed in DP thymocytes (Fig. 2*A*). DP and SP thymocytes from Lck-Cre-hM3Dq–PLCβ1 mice exhibited weak calcium responses to clozapine stimulation (Fig. 2*B*). Surprisingly, even though peripheral hM3Dq/β1 T cells expressed hM3Dq and PLCβ1, these T cells showed no calcium responses to clozapine stimulation (Fig. 2 *C* and *D*). However, we found that hM3Dq/β1 T cells prestimulated with anti-CD3 and anti-CD28 mAbs and cultured for 3 d and then incubated with either IL-2 or IL-7 for 2 d showed markedly increased calcium responses to clozapine stimulation (Fig. 2*E*). This might be attributable to the higher PLCβ1 and hM3Dq expression in T cells stimulated with anti-TCR/CD28 reactive mAbs and IL2 (Fig. 2*F* and *SI Appendix*, Fig. S2*F*). Indeed, the preactivated T cells with more highly expressed hM3Dq and PLCβ1 exhibited larger calcium responses compared to those with lower hM3Dq and PLCβ1 expression (*SI Appendix*, Fig. S2*G*). Thus, TCR/CD28 stimulation and IL-2 (or IL-7) incubation led to larger calcium increases in hM3Dq/β1 T cells in response to clozapine stimulation most likely due to the increased expressions of hM3Dq and PLCβ1. These preactivated hM3Dq/β1 T cells were used in upcoming experiments to study the functional consequences of activating the muscarinic receptors in primary T cells. Stimulation of hM3Dq or M1 receptors that could couple to PLCβ1 also induced larger and more sustained calcium and pErk responses (Fig. 2 *G* and *H* and *SI Appendix*, Fig. S2 *H–K*). In summary, activation of hM3Dq which was able to couple to PLCβ1 induced substantial calcium increases and phosphorylated ERK responses in preactivated primary T cells.

In Jurkat cells, muscarinic receptors bypass PTK-dependent TCR signaling pathways (6–8). Similarly, the Src kinase inhibitor PP2 suppressed induced phosphotyrosine, pERK, and calcium responses by anti-CD3 stimulation but only showed minimal effects in pERK and calcium responses to clozapine stimulation in hM3Dq/β1 T cells (Fig. 2 *I*–*M*). Thus, these results suggested that muscarinic receptors can bypass classical PTK-dependent TCR pathways in primary T cells.

### Stimulation of hM3Dq Induces IFN-γ but Reduces IL-2 in TCR-Stimulated hM3Dq/β1 T Cells.

**Fig. 3.**
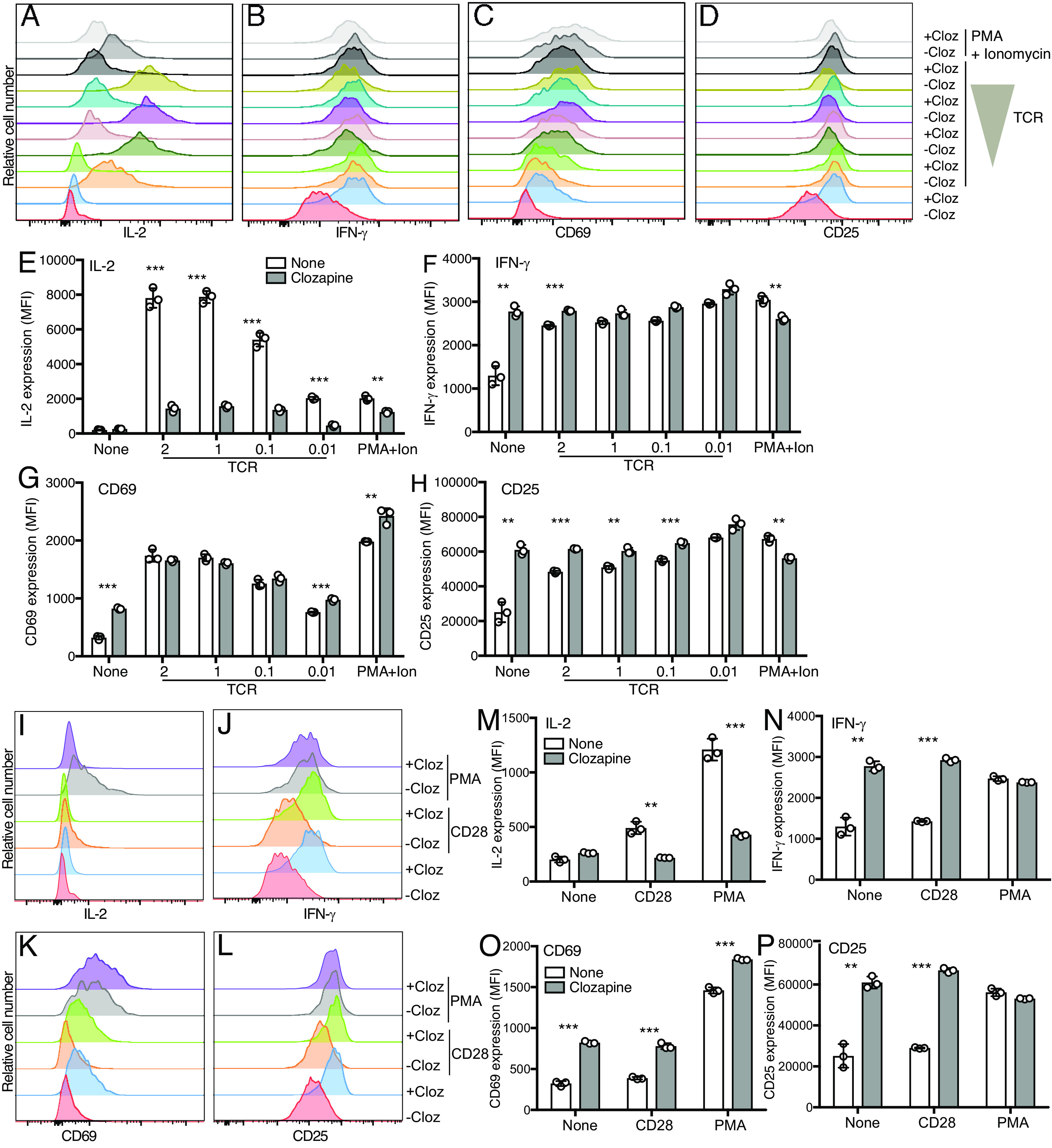
Stimulation of hM3Dq induces IFN-γ but reduces IL-2 in TCR-stimulated hM3Dq/β1 T cells. CD4 T cells from LckCre-hM3Dq–PLCβ1 mice were activated with anti-CD3 (1 μg/mL) + anti-CD28 (2 μg/mL) and IL-2 (40 U/mL) for 3 d and then cultured with IL-2 (40 U/mL) for 2 d. (*A–H*) hM3Dq/β1 CD4 T cells in response to 10 μM clozapine (cloz) and/or TCR stimulation (2, 1, 0.1, or 0.01 μg/mL anti-CD3 + 2 μg/mL anti-CD28) and/or PMA (50 ng/mL) + ionomycin (ion, 1 μM) for 16 h. (*A–D*) Overlaid histograms of IL-2, IFN-γ, CD69, and CD25 expression in hM3Dq/β1 CD4 T cells. (*E–H*) Bar chart shows MFI of IL-2, IFN-γ, CD69, and CD25 expression in hM3Dq/β1 CD4 T cells. (*I–P*) hM3Dq/β1 CD4 T cells in response to 10 μM clozapine and/or PMA (50 ng/mL) and 2 μg/mL anti-CD28 for 16 h. (*I–L*) Overlaid histograms and (*M–P*) bar charts showing IL-2, IFN-γ, CD69, and CD25 expression in hM3Dq/β1 CD4 T cells. Data are representative of three independent experiments. *P < 0.05, **P < 0.005, ***P < 0.0005.

Stimulation of muscarinic receptors with clozapine did not induce the expression of IL-2, but potently induced IFN-γ, CD69, and CD25 expression in hM3Dq/β1 CD4 T cells (Fig. 3 *A*–*H*). Interestingly, addition of clozapine to TCR-stimulated T cells resulted in significantly reduced IL-2 responses but did not change or in some cases even increased IFN-γ, CD69, and CD25 expression in hM3Dq/β1 CD4 T cells (Fig. 3 *A*–*H*). We also observed similar effects in hM3Dq/β1 CD8 T cells (*SI Appendix*, Fig. S3 *A–H*). Previously, costimulation with PMA plus carbachol was reported to lead to increased IL-2 production in hM1+ Jurkat cells (7). However, clozapine stimulation combined with PMA or CD28 did not induce IL-2, nor did it increase IFN- γ and CD25 responses in primary hM3Dq/β1 T cells compared with clozapine alone (Fig. 3 *I*–*P*). Stimulation with clozapine and PMA led to increased CD69 expression compared with clozapine alone (Fig. 3 *K* and *O*). Similar to TCR stimulation, addition of clozapine also resulted in reduced IL-2, but not IFN-γ in CD28- or PMA-stimulated hM3Dq/β1 T cells (Fig. 3 *I*–*P*). In summary, stimulation of the hM3Dq receptor with clozapine with or without costimulation (PMA or anti-CD28) induces IFN-γ, CD69, and CD25. However, stimulation with clozapine does not substantially increase IL-2 and, instead, leads to decreased IL-2 expression in TCR costimulated hM3Dq/β1 T cells.

### Stimulation of hM3Dq Induces Nuclear Translocation of NFAT and NFκB and Activates AP-1 in hM3Dq/β1 T Cells.

**Fig. 4.**
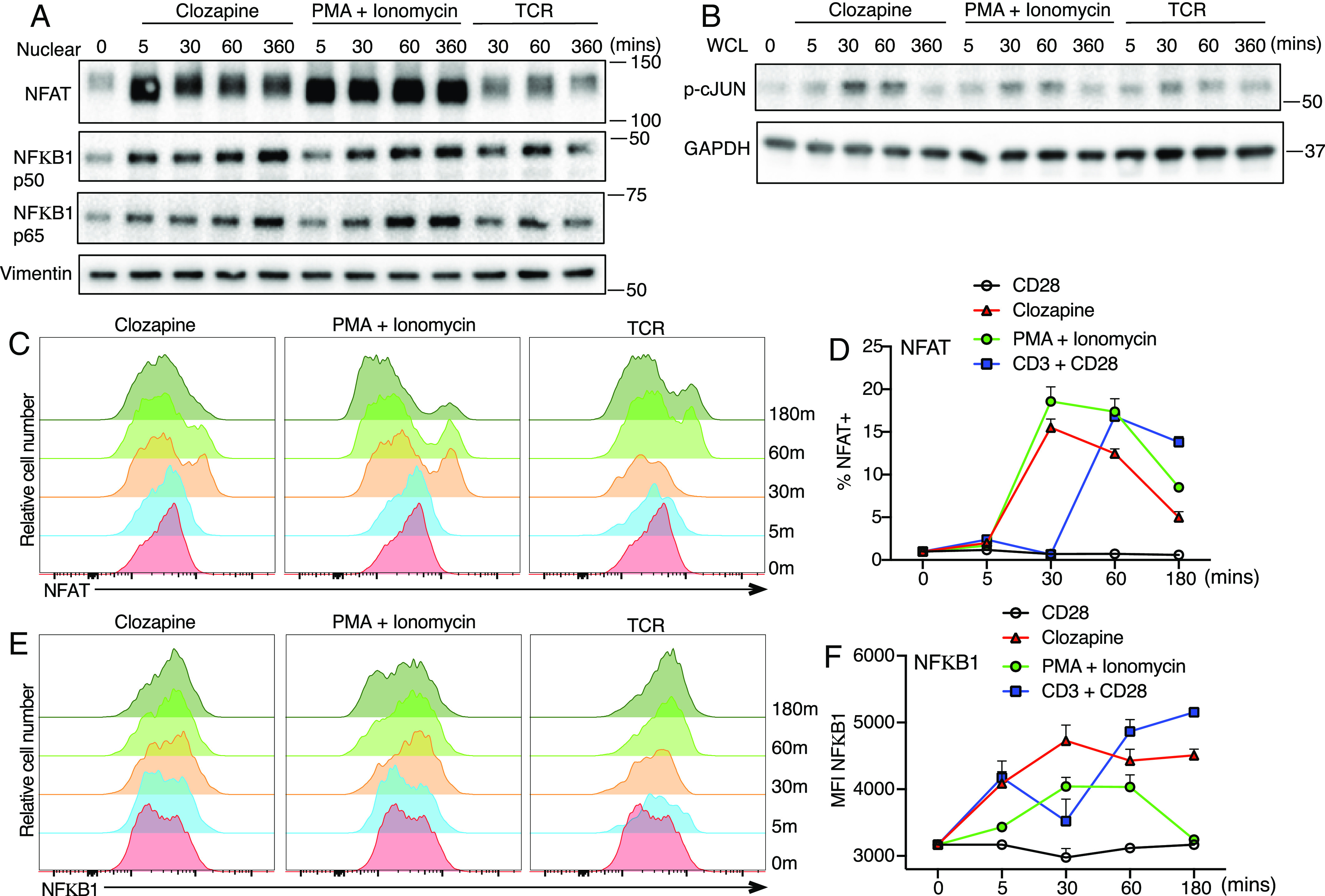
Stimulation of hM3Dq induces nuclear translocation of NFAT and NFκB in hM3Dq/β1 T cells. (*A*) Immunoblot analysis of NFAT, NFκB1 p50, and p65 in the nuclear extracts of hM3Dq/β1 CD4 T cells stimulated with clozapine, plate-bound anti-CD3 + anti-CD28 (TCR), or PMA + ionomycin over time. Vimentin, loading control. (*B*) Immunoblot analysis of p-cJun in whole-cell lysates of hM3Dq/β1 CD4 T cells over time after stimulation. Gapdh, loading control. (*C* and *D*) Overlaid histograms and charts show NFκB1 expression in the nuclei of hM3Dq/β1 CD4 T cells. (*E* and *F*) Overlaid histograms and charts show NFAT expression in the nuclei of hM3Dq/β1 CD4 T cells. Data are representative of three independent experiments. ***P < 0.0005.

Since nuclear factor of activated T cells (NFAT) family members, nuclear factor- κB (NFκB), and activator protein 1 (AP-1) bind to the promoter and upstream regions relative to the transcription start site of IL-2, we determined whether activation of hM3Dq receptors induced activation of these transcription factors. We found that clozapine induced nuclear translocation of NFAT and NFκB and also phosphorylated c-Jun (an AP-1 subunit) in preactivated hM3Dq/β1 CD4 T cells (Fig. 4 *A* and *B*). Flow cytometry of nuclear staining for NFAT and NFκB1 consistently confirmed that stimulation of hM3Dq greatly induced nuclear translocation of NFAT and NFκB, comparably to PMA + ionomycin or to TCR stimulation, albeit with somewhat altered kinetics (Fig. 4 *C*–*F*). In summary, stimulation of muscarinic receptors with PLCβ1 induced strong nuclear translocation of NFAT and NFκB and phorphorylated c-Jun, an indication of AP-1 activation, in hM3Dq/β1 T cells.

### Stimulation of hM3Dq Results in Reduced IL-2 mRNA Stability Measured by 3′UTR Activity of IL-2 mRNA in TCR-Prestimulated hM3Dq/β1 CD4 T Cells.

**Fig. 5.**
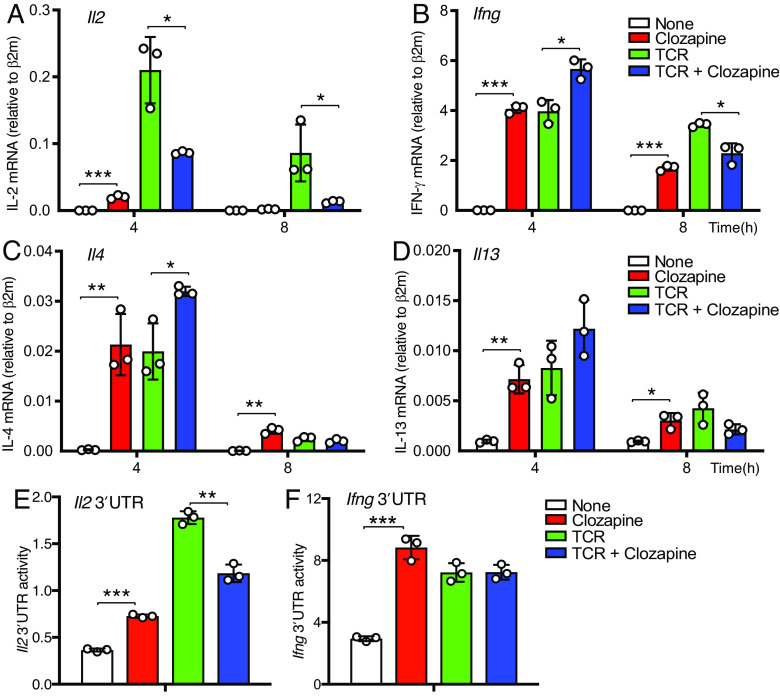
Stimulation of hM3Dq results in reducing IL-2 mRNA stability measured by 3′ untranslated region (UTR) activities of IL-2 mRNA in TCR-stimulated hM3Dq/β1 CD4 T cells. (*A–D*) Relative expression of IL-2, IFN-γ, IL-4, and IL-13 mRNA in hM3Dq/β1 CD4 T cells. (*E* and *F*) CD4 T cells from LckCre-hM3Dq–PLCβ1 mice were transfected with dual-luciferase reporter plasmid containing (*E*) 3′UTR IL-2 or (*F*) 3′UTR IFN-γ mRNA. Bar chart shows (*E*) 3′UTR activities of IL-2 or (*F*) IFN-γ mRNA in hM3Dq/β1 CD4 T cells 4 h after stimulation. Data are representative of two independent experiments. *P < 0.05, ***P < 0.0005.

To determine whether clozapine stimulation differentially affects cytokine mRNA expression, we measured various cytokine mRNA at 4 and 8 h after stimulation. Compared to stimulation of the TCR, stimulation of muscarinic receptors resulted in smaller increases in IL-2 mRNA, but larger relative increases in IFNγ, IL-4, and IL-13 mRNA in hM3Dq/β1 CD4 T cells (Fig. 5 *A*–*D*). Thus, these results are consistent with IL-2 and IFNγ protein expression detected by intracellular staining (Fig. 3 *A, B*, *E*, and *F*), and cells stimulated via muscarinic receptors exhibited greatly reduced IL-2 but increased IFNγ, IL-4, and IL-13 mRNA expression in TCR-prestimulated hM3Dq/β1 CD4 T cells at 4 h after stimulation (Fig. 5 *A*–*D*).

IL-2 mRNA stability is highly regulated by RNA-binding proteins that target the 3′ untranslated mRNA segment (18). To determine whether the differential effects of clozapine on IL-2 versus IFN-γ expression in TCR-stimulated hM3Dq/β1 CD4 T cells might be due to a differential RNA stability that was governed by the 3′ untranslated region, we transfected the cells with the Psicheck2 plasmid in which *Renilla* luciferase reporter transcript contains 3′UTRs of either IL-2 or IFN-γ mRNA and performed luciferase assays (Fig. 5 *E* and *F*). Our results showed that the reporter carrying a 3′UTR derived from IL-2 but not the IFN-γ mRNA was strongly reduced in the TCR plus clozapine stimulation compared to TCR stimulation alone in hM3Dq/β1 CD4 T cells (Fig. 5 *E* and *F*). Thus, stimulation of muscarinic receptor resulted in an active reduction in IL-2 mRNA stability by reducing 3′UTR activities of IL-2 mRNA but showed no effects in 3′UTR activities of IFN-γ mRNA in TCR-stimulated hM3Dq/β1 CD4 T cells. In summary, addition of clozapine stimulation of the hM3Dq results in reduced 3′UTR activities of IL-2 mRNA, which can at least partially explain the potent decrease in IL-2 mRNA and protein expression in clozapine plus TCR-stimulated hM3Dq/β1 CD4 T cells.

### Stimulation of hM3Dq Reduces Activation of pAKT Pathways in hM3Dq/β1 T Cells.

**Fig. 6.**
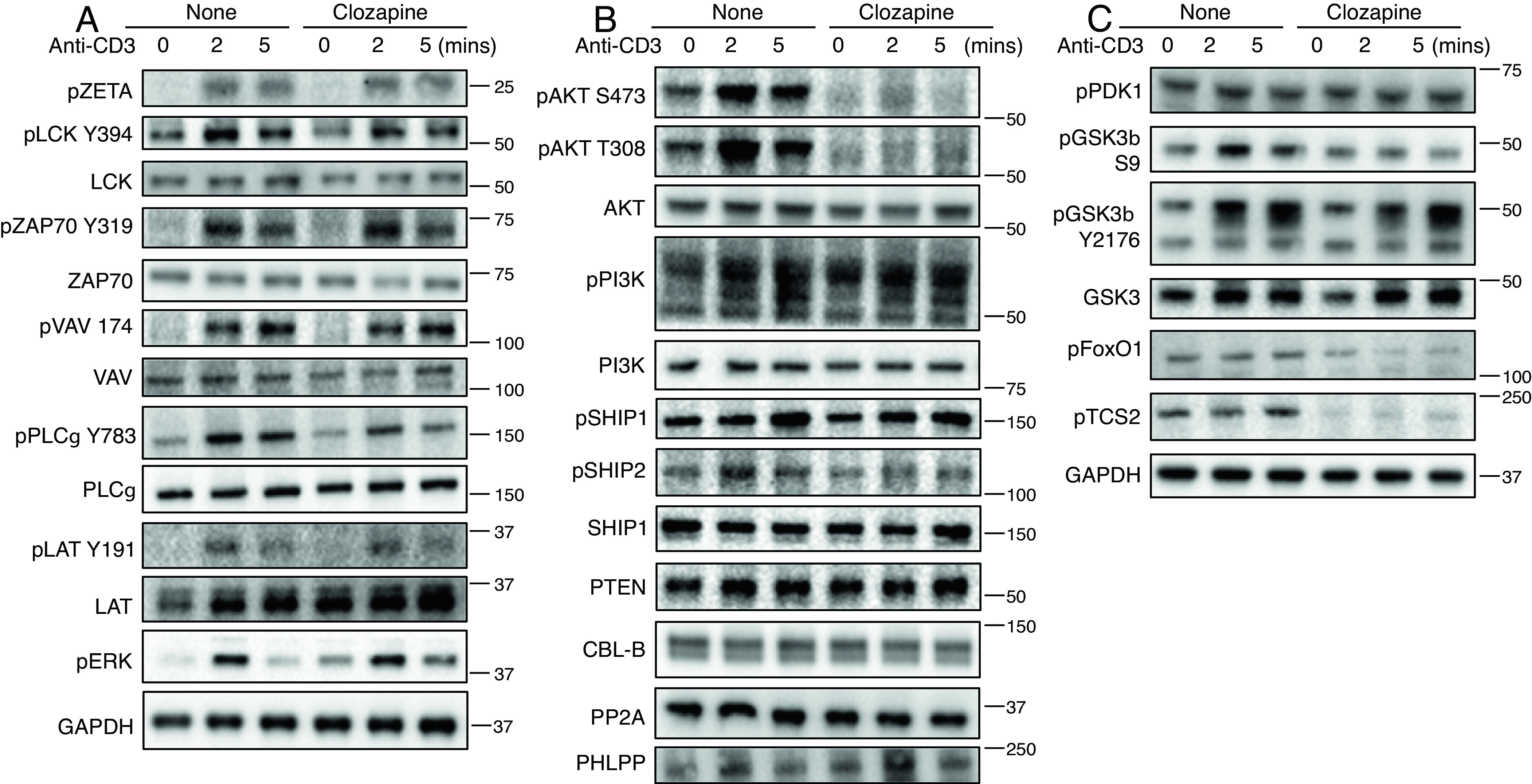
Stimulation of hM3Dq reduces pAKT pathways in hM3Dq/β1 T cells. hM3Dq/β1 CD4 T cells stimulated with clozapine for 15 min, then with anti-CD3 (1 μg/mL) followed by cross-linking with 20 μg/mL anti-Armenian hamster IgG for 2 and 5 min. (*A*) Immunoblot analysis of phosphorylation of total TCR proximal signaling molecules in hM3Dq/β1 CD4 T cells. (*B*) Immunoblot analysis of PI3K–pAKT pathways and negative regulators of PI3K–pAkt in hM3Dq/β1 CD4 T cells. (*C*) Immunoblot analysis of phosphorylation of Akt substrates in hM3Dq/β1 CD4 T cells. Gapdh, loading control. Data are representative of two independent experiments.

Since clozapine greatly reduced IL-2 expression in TCR-stimulated T cells, we determined whether clozapine negatively impacted proximal TCR signaling pathways in hM3Dq/β1 CD4 T cells. Interestingly, our results showed that costimulation of hM3Dq did not affect major proximal TCR signaling events that induce tyrosine phosphorylation of various tyrosine phosphoproteins downstream of the TCR (Fig. 6*A*). However, since signaling through the PI3K on T cells can modulate the half-life of a select subset of cytokine mRNAs, such as IL-2 via RNA-binding proteins (RBPs) (19, 20), we assessed whether clozapine affects PI3K signaling pathway in stimulated T cells. Activation of PI3K leads to production of PIP3 at the plasma membrane (21, 22). Cytosolic AKT is recruited to the membrane and engages PIP3 through its PH domain and is phosphorylated at T308 and S473 by PDK1 and mTORC2, respectively (21, 23). Stimulation of muscarinic receptors greatly reduced activation of pAKT both at S473 and T308 in anti-CD3-stimulated hM3Dq/β1 T cells (Fig. 6*B*). Clozapine only showed small decreases or no effects in phosphorylated PI3K and negative regulators of PI3K (24–26), such as pSHIP1, pSHIP2, and PTEN (Fig. 6*B*). Total protein expressions of the E3 ubiquitin ligase Cbl-b, a negative regulator of TCR signaling, and phosphatases PP2A and PHLPP, negative regulators of activated AKT (24–26), were also not affected by clozapine (Fig. 6*B*). In conclusion, costimulation of hM3Dq did not affect tyrosine phosphorylation of major TCR signaling events but greatly reduced the phosphorylation and by inference the activation of pAkt.

AKT phosphorylates targets on Ser and Thr residues, primarily within a minimal consensus recognition motif of RXXS*/T* (where X is any amino acid and * denotes a preference for large hydrophobic residues) to often inhibit the function of the given target. AKT activation can promote cell survival, proliferation, growth, and changes in cellular metabolic pathways through its numerous downstream targets. Three best-established downstream targets of AKT include glycogen synthase kinase 3 (GSK-3), the forkhead box O transcription factor (FoxO), and tuberous sclerosis complex 2 (TSC2) (21). Consistent with decreased pAKT, clozapine also reduced pAKT substrates, including pGSK3b at pSer9, pFoxO1, and pTCS2 (Fig. 6*C*). In contrast, pGSK3b Y2176, which is not an AKT substrate, and phosphorylation of phosphoinositide-dependent protein kinase 1 (PDK1), which phosphorylates AKT at T308, showed no substantial impact by clozapine treatment. Thus, stimulation of muscarinic receptors strongly reduced activation of pAKT pathways and its downstream molecules, which may explain the effect on IL-2 production in hM3Dq/β1T cells.

### Inhibition of PI3K Reduces IL-2 Production in TCR-Stimulated hM3Dq/β1 CD4 T Cells.

**Fig. 7.**
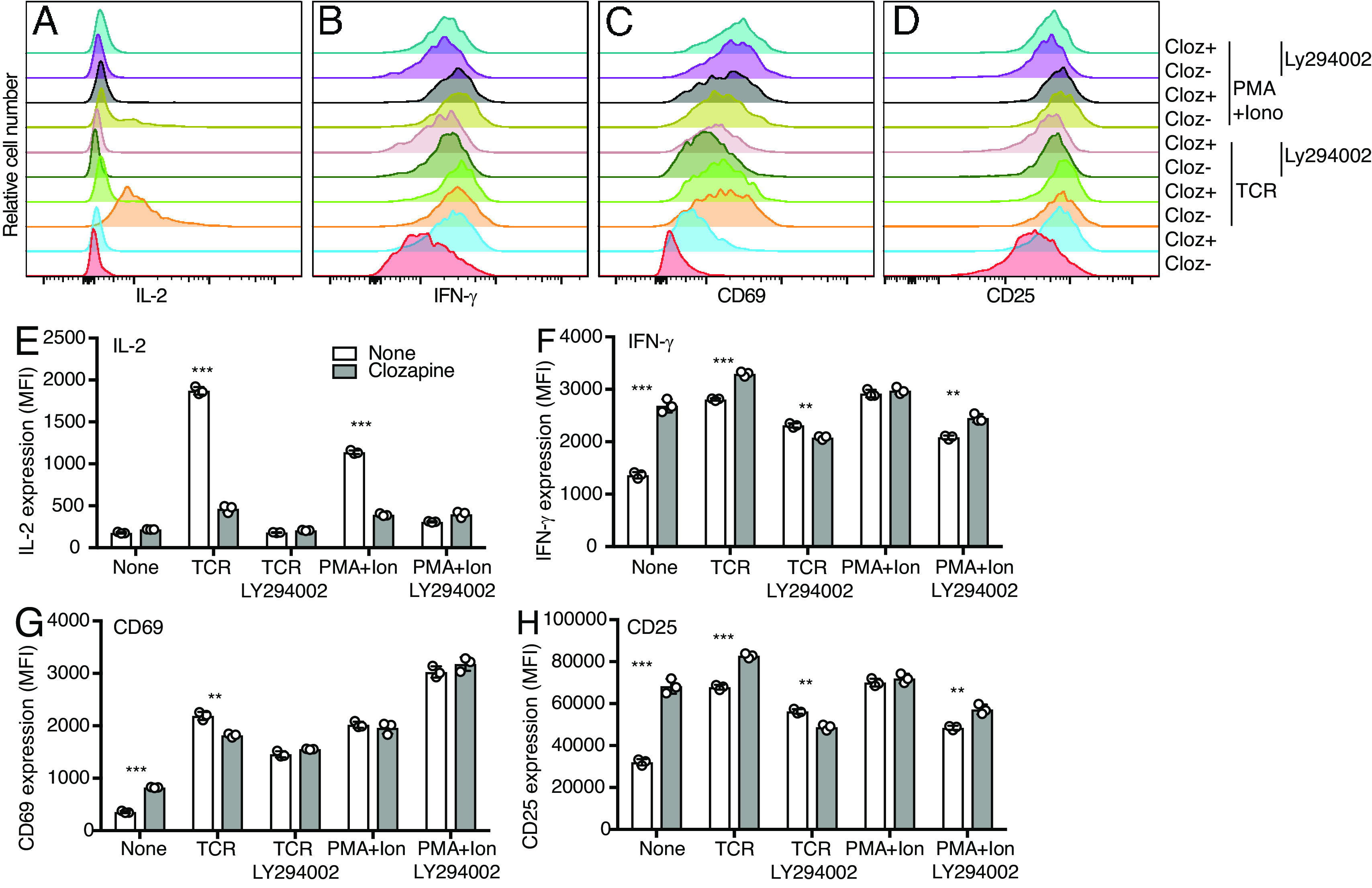
Inhibition of PI3K reduces IL-2 production in TCR-stimulated hM3Dq/β1 CD4 T cells. hM3Dq/β1 CD4 T cells in response to clozapine (cloz), TCR stimulation, and PMA + ionomycin (PMA+Ion) in the presence of PI3K inhibitor (Ly294002) for 16 h. (*A–D*) Overlaid histograms of IL-2, IFN-γ, CD69, and CD25 expression in hM3Dq/β1 CD4 T cells. (*E–H*) Bar chart shows MFI of IL-2, IFN-γ, CD69, and CD25 expression in hM3Dq/β1 CD4 T cells. Data are representative of two independent experiments.

Since the PI3K pathway is involved in AKT activation, we asked whether inhibition of PI3K would mimic the effect of muscarinic receptor stimulation in selectively blocking IL-2 but not IFNγ, CD69, or CD25 expression in TCR-stimulated cells. A PI3K inhibitor (Ly294002) was used to determine whether pAKT pathway is critical for IL-2 production. Indeed, we found that the PI3K inhibitor Ly294002 strongly reduced IL-2 expression in TCR or PMA plus ionomycin-stimulated T cells that were preactivated via TCR stimulation followed by culture in IL-2 or IL-7 (Fig. 7 *A*–*H*). In contrast, the PI3K inhibitor showed much less effect on IFN-γ, CD65, and CD25 expression compared to IL-2 expression in the preactivated T cells (Fig. 7 *A*–*H*). In conclusion, the PI3K/pAkt pathway is critical for IL-2 production in previously stimulated T cells.

## Discussion

Studying the functions of muscarinic receptors, which are largely PTK independent, might inspire new therapies for bypassing states of T cell unresponsiveness, such as T cell anergy and T cell exhaustion, or the negative regulatory impact of coinhibitory receptors that depend on tyrosine phosphorylation pathways. However, we found that stimulation of muscarinic receptors M1 and hM3Dq showed much lower-than-anticipated calcium responses in primary T cells compared to those in Jurkat cells. Transduction of PLCβ1, a predominant PLCβ isoform expressed in Jurkat but not in primary T cells (13, 15), in preactivated T cells led to increased muscarinic receptor and PLCβ1 expression and greatly amplified T cell calcium increases and phosphorylated ERK in response to stimulation of muscarinic receptors. Unexpectedly, whereas stimulation of muscarinic receptors that couple to PLCβ1 via Gq proteins induced IFN-γ, CD69, and CD25 as well as some other cytokines, it surprisingly did not lead to substantial induction of IL-2 in hM3Dq/β1 T cells. Importantly, stimulation of muscarinic receptors coupled to PLCβ1 on previously activated T cells even reduced IL-2 expression in response to TCR stimulation of hM3Dq/β1 T cells. This reduction in IL-2 expression could not be accounted for by a reduction in the activation of NFAT, NFκB, or phosphorylation of AP-1. We noted that the relatively selective effect on IL-2 may be attributable, in part, to a reduction in the 3′UTR activity of IL-2 mRNA that could lead to reduced IL-2 mRNA stability.

How clozapine stimulation of the muscarinic HM3Dq receptor led to inhibition of IL-2 3′UTR was not entirely clear. However, we found that clozapine stimulation of hM3Dq/β1 T cells resulted in a reduction in pAKT and its downstream pathway signaling molecules. This could impact IL-2 production in hM3Dq/β1-stimulated T cells based on a negative regulatory effect on an inhibitor of the PI3K pathway. Indeed, we found that a potent PI3K inhibitor reduced IL-2 production in TCR-stimulated hM3Dq/β1 CD4 T cells. Together, these findings suggest that the pAKT pathway is critical for IL-2 production in previously activated T cells. Future studies in designing CAR T cells or other immune therapies might need to consider whether a PI3K/pAKT increase and subsequent IL-2 production by these cells would be desirable. We would predict, but have not studied here, the ability of hM3Dq receptor to resist the negative regulatory impact of ITIM and other inhibitory constraints targeting tyrosine phosphorylation pathways. Still, attention needs to consider situations where IL-2 production is important. The insights gained from these studies will be informative about the key aspects of pAKT and IL-2 production by T cells.

Previous studies have supported our results showing that the PI3K/pAKT pathway is critical for IL-2 induction by using the PI3K inhibitors LY294002, IC87114, and wortmannin (27, 28). LY294002 and wortmannin blocked IL-2 production and T cell proliferation in antigen-stimulated or anti-CD3-stimulated T cells (27, 28), thereby supporting a role for PI3K signaling in the IL-2 production. However, these PI3K inhibitors also reduced the production of other cytokines, including IFN- γ, IL-4, IL-17, and TNF-a in anti-CD3-stimulated naïve T cells (27, 28). The different effects of LY294002 on IFN-γ production between previous studies and the effects of muscarinic receptors in this study were probably due to the two separate activation/differentiation states of T cells that were studied, i.e., naïve T cells versus previously stimulated T cells (preactivated via TCR stimulation followed by IL-2 or IL-7), respectively. These results suggest that IFN-γ production in previously stimulated T cells is less sensitive to PI3K inhibitors.

The impact of the PI3K/pAKT pathway in promoting IL-2 production was a bit unexpected based on classical studies of the IL-2 upstream promoter. However, more recent studies suggest that the PI3K/AKT pathway promotes IL-2 mRNA stability by mediating effects on RBPs. RBPs, which determine mRNA maturation, localization, stability, and translation, exhibit binding activity through the recognition of adenylate and uridylate–rich elements (AREs) or U-rich elements that are often found in the 3′UTR of their target mRNAs (22, 23). Stimulation of hM3Dq led to reduced 3′UTR activities of IL-2 mRNA and subsequently IL-2 mRNA and protein expression in TCR-stimulated hM3Dq/β1T cells. In contrast, 3′UTR activities of IFN-γ mRNA and protein expression were not affected by clozapine stimulation of TCR-stimulated hM3Dq/β1 T cells. Together, these results suggest that stimulation of hM3Dq influences specific subsets of RNA-binding proteins involved in regulating IL-2 mRNA stability. Binding of YB-1 and nucleolin to the 5′-UTR and binding of HuR and NF-90 to the 3′-UTR have been implicated in the PI3K/CD28-mediated stabilization of the IL-2 mRNA stability (20, 29–32). Interestingly, HuR-deleted CD4+ T cells have strikingly increased IL-2, with no change in IFN-γ expression (33, 34). Future studies should focus on characterizing these RNA-binding proteins, especially HuR, in influencing IL-2 but not IFN-γ mRNA stability in stimulated hM3Dq/β1 T cells. Such studies could impact the potential use of the muscarinic receptor approach described here in future immunotherapy.

## Methods

### Mice.

Mice, 8 to 12 wk of age, of the strains C57BL/6, hM3Dq [B6N;129-Tg(CAG-CHRM3*, mCitrine)1Ute/J, Strain #:**026220**], and Lck-Cre [B6.Cg-Tg(Lck-cre)548Jxm/J, Strain #:**003802**] were purchased from The Jackson Laboratory. All mice were housed in the specific pathogen-free facilities at the University of California, San Francisco, and were treated according to protocols that were approved by the UCSF Animal Care Ethics and Veterinary Committees, and in accordance with NIH guidelines.

The inducible PLCβ1-mCherry mouse strain was generated by gene targeting in C57BL/6 ES cells similar to previous studies (35, 36). PLCβ1-IRES-mCherry was cloned into the CTV plasmid (Addgene plasmid # 15912; http://n2t.net/addgene:15912; RRID:Addgene_15912). The CAG-PLCβ1-mCherry allele allows Cre recombinase–inducible expression of a CAG promoter–driven PLCβ1-IRES-mCherry. Following Cre-mediated removal of an upstream floxed-STOP cassette, expression of PLCβ1 and mCherry fluorescent protein is observed. We successfully generated seven ES cell clones with properly inserted PLCβ1 in the Rosa 26 locus, confirmed by long-range PCR. We used PCR primers 5′ and 3′ of the Rosa26 arm of genomic DNA isolated to detect 2 representative ES clones (11 and 94) containing PLCβ1-IRES-mCherry knocked into the Rosa26 locus. PLCβ1 expression in T cells was monitored by the expression of mCherry separated from the PLCβ1 by an IRES sequence. Two ES clones (11 and 94) were selected to be injected into embryos to generate chimeric mice. Inducible PLCβ1 knock-in chimeras were crossed with hM3Dq × Lck-Cre mice to generate mice in which only T cells expressed both hM3Dq and PLCβ1 to study muscarinic receptor and PLCβ1 pathways in T cells.

For PLCβ1 genotyping, an mCherry forward primer GACCGCCAAGCTGAAGGTGACC and a reverse primer GCGCGTTCGTACTGTTCCACGA were used. The desired amplicon within mCherry is 525 bp. Inducible PLCβ1 knock-in mice were crossed with Lck-Cre × hM3Dq mice to generate LckCre-hM3Dq–PLCβ1 mice in which only T cells expressed both hM3Dq and PLCβ1 to study muscarinic receptor and PLCβ1 pathways in T cells.

### Lentivirus Transduction.

M1 or hM3Dq was cloned into the pHR backbone plasmid under the control of the *EF1A* promoter. A C-terminal P2A self-cleaving peptide followed by mCherry was incorporated to assess transduction efficiency and expression levels. The packaging vector pCMV dR8.91, envelope vector pMD 2.G, and pHR M1 or hM3Dq constructs were transiently cotransfected into LX-293T cells with TransIT-LT1 reagent (Mirus Bio Mio2300). Supernatants containing virus particles were collected 48 h after transfection, filtered, and pelleted before use. The virus particles were resuspended in PBS and stored at –80 °C. The supernatants were collected and filtered 48 h after transfection, and the Jurkat cells were transduced by centrifugation (1,200 g, 45 min).

### Retroviral Transduction.

M1 or hM3Dq was cloned into the MSCV backbone plasmid [MSCV-IRES-GFP was a gift from Tannishtha Reya (Addgene, plasmid #20672; http://n2t.net/addgene:20672; RRID:Addgene_20672)]. A flag-tag sequence was added at the C terminus of M1 to monitor M1 expression level. PLCβ1 was cloned into the MSCV backbone plasmid at the EcoRI and XhoI sites [MSCV-IRES-mCherry FP was a gift from Dario Vignali (Addgene, plasmid #52114; http://n2t.net/addgene:52114; RRID:Addgene_52114)]. The pcL-Eco and MSCV vectors were cotransfected into Phoenix-Eco packaging cell line using Lipofectamine 2000 (Invitrogen). Supernatants containing virus particles were collected 48 h and 72 h after transfection. The virus particles were concentrated using a Retro-X concentrator (Takara, Cat 631455) resuspended in PBS and stored at –80 °C. Retroviral transduction of T cells were stimulated with plate-bound anti-CD3 (2C11, 1 μg/mL), soluble anti-CD28 (37.51, 2 μg/mL), and IL-2 (40 U/mL) in culture medium (RPMI supplemented with L-glutamine/streptomycin and 10% fetal bovine serum) in 96-well round-bottom plates at 37 °C in 5% CO2 in a 6-well plate for 36 h. Lipofectamine was added to the thawed retroviral supernatant at a concentration of 8 μg/mL and incubated for 30 min at room temperature. Prestimulated T cells were transduced with the retroviral supernatant containing Lipofectamine by centrifugation (1,200 g, 25 min). Two days following transduction, viral supernatant was replaced with culture media containing IL-2 (40 U/mL). T cells were cultured with IL-2 for at least two additional days.

### T Cell Isolation and In Vitro T Cell Assays.

Peripheral CD3+ or CD4+ or CD8+ T cells were negatively enriched by conjugated magnetic beads using biotinylated antibody depletion mixtures and antibiotin microbeads (Miltenyi Biotech) after Fc blocking with rat serum. For the isolation of CD3+ T cells from the periphery, cells were stained with biotinylated antibodies to CD45R, CD11b, CD24, CD8a, CD49b, Ter119, CD19, and CD11c. For the isolation of CD4 T cells, CD8a-biotin was added to the above antibody mixture. Purified T cells were cultured with plate-bound anti-CD3 (2C11, 1 μg/mL), soluble anti-CD28 (37.51, 2 μg/mL), and IL-2 (40 U/mL) in medium (RPMI supplemented with L-glutamine/streptomycin and 10% fetal bovine serum) in 96-well round-bottom plates at 37 °C in 5% CO2 for 3 d. Activated T cells were washed with PBS and cultured in IL-2 (40 U/mL) or IL7 (10 ng/mL) for 2 more days and washed with culture media. Prestimulated T cells were stimulated with clozapine (10 μM) and/or anti-CD3 (2, 1, 0.1, or 0.01 μg/mL) and/or anti-CD28 (2 μg/mL) and/or PMA (50 ng/mL) and ionomycin (1 μM).

### Flow Cytometry.

Purified T cells were stimulated with plate-bound anti-CD3 (2C11, 1 μg/mL), soluble anti-CD28 (37.51, 2 μg/mL), and IL-2 (40 U/mL) in culture medium in 96-well round-bottom plates at 37 °C in 5% CO2 for 3 d. Activated T cells were washed with PBS and cultured in IL-2 (40 U/mL) or IL-7 (10 ng/mL) for 2 more days and washed with culture medium. Preactivated T cells were restimulated with clozapine (10 μM) and/or anti-CD3 (2, 1, 0.1, or 0.01 μg/mL) and/or anti-CD28 (2 μg/mL) and/or PMA (50 ng/mL) and ionomycin (1 μM) overnight. The cells were incubated with anti-CD16/32 at 5 μg/mL for 20 min on ice to block Fc receptors. IL-2- and IFN-γ secreting T cells were identified using the IL-2 and IFN-γ secretion assays (Miltenyi Biotech) according to the instructions of the manufacturer. Flow cytometry analysis was performed to identify mean fluorescent expression levels (MFI) of CD69, CD25, IL-2, and IFN-γ of T cells with anti-CD4 BV395 (BD), CD8 BV737 (BD), CD25 percpCy55 (Tonbo), CD69 BV605 (BioLegend), IL-2-BV421 (BD), and IFN-γ-APC (Miltenyi Biotech) antibody staining. Anti-HA APC (BioLegend) and anti-Flag APC (BioLegend) antibodies were used to detect hM3Dq and M1 expression, respectively. Dead cells were excluded using the live/dead-fixable Near-IR death cell stain kit (Invitrogen, L34976 A).

T cells were stimulated with clozapine (10 μM) or PMA (50 ng/mL) and ionomycin (1 μM) or anti-CD3 (1 μg/mL) and/or anti-CD28 (2 μg/mL) for 5, 30, 60, or 360 min. Isolation of primary T cell nuclei for flow cytometry was done as previously described (37). For nuclear staining, antibodies against NFAT1 (Cell signaling, #5861) or NFκB1 p65 (Cell Signaling, #8242) were used. Antibody against Alex647-anti rabbit IgG was used as a secondary antibody to detect NFAT1 and NFκB1 p65.

### Calcium Responses.

Jurkat cells or primary mouse cells were surface-stained and loaded with Indo-1 dye (Invitrogen) for 30 min at 37 °C in RPMI supplemented with 5% fetal bovine serum and HEPES. After loading with Indo-1, cells were washed and analyzed by flow cytometry (LSR Fortessa with a UV laser) at 37 °C, stimulated with carbachol (M1 agonist, 500 μM) or clozapine (hM3Dq agonist, 10 μM) and ionomycin (1 μM) or 1 μg/mL anti-TCR (C305) for Jurkat cells or anti-CD3 for mouse cells over time. Calcium increases were monitored as the ratio of Indo-1 (blue) and (violet) and displayed as a function of time.

### Immunoblot Analyses.

Peripheral T cells were washed with PBS and resuspended at 2 × 10^6^ cells/100 μL culture media and incubated for 30 min at 37 °C. The cells were left unstimulated or were stimulated with clozapine (hM3Dq agonist, 10 μM) for 15 min, and then soluble anti-CD3 (2C11, final concentration 1 μg/mL) and secondary antibody (goat anti-Armenian Hamster IgG, final concentration 20 μg/mL) for 2 and 5 min, respectively. Cells were lysed by the addition of 25 μL of 6× lysis buffer (6% NP-40, 12 mM NaVO4, 60 mM NaF, 30 mM EDTA, 12 mM PMSF, 60 μg/mL aprotinin, 6 μg/mL pepstatin, and 6 μg/mL leupeptin). The lysates were placed on ice and centrifuged at 13,000 g to pellet cell debris. 6× loading dye was added to the supernatant. Samples were heated in sand at 100 °C for 5 min. The supernatants were run on NuPAGE 4–12% Bis-Tris protein gels (Thermo Fisher) and transferred to PVDF membranes.

Nuclear isolation for western blotting used a cell fractionation kit (Cell signaling, 9038S) per manufacturer’s instruction. Stimulated T cells (2 × 10^6^ cells in 100 μL culture media) were cultured with clozapine (10 μM) or PMA (50 ng/mL) + ionomycin (1 μM), or plate-bound anti-CD3 (1 μg/mL) + anti-CD28 (2 μg/mL) over time. Antibodies against NFAT1 (#5861), NFκB1 p105/p50 (#12540), NFκB1 p65 (#8242), and vimentin (#5741) from Cell Signaling were used.

The samples were run on NuPAGE 4–12% Bis-Tris protein gels (Thermo Fisher) and transferred to PVDF membranes. The membranes were blocked with TBS-T buffer containing 3% BSA for 2 h at room temperature, and then probed with primary antibodies. Primary antibodies for the following specificities were used: LCK (1F6), ZAP70 (1E7.2), PLCβ1 (Abclonal, A1971), phospho-GSK-3β (pY216, BD, #612312), phosphor-PLCγ (Tyr 783, Invitrogen, #44-696G), PHLPP (ThermoFisher, 22789-1-AP), PLCγ (EMD, #05-163), phospho-zeta (pY142, BD, cat 558402), phosphorylated VAV Tyr174 (Abcam, #ab47282), and VAV (Transduction Lab, #v13230/l4). Other antibodies from Cell Signaling included PP2A C Subunit Antibody (#2038), phospho-c-JUN (Ser73) (#3270), Phospho-PDK1 (Ser241) (#3438), Phospho-GSK-3β (Ser9) (#9336), Phospho-FoxO1 (Ser256) (#9461), Phospho-mTOR (Ser2481) (#2974), Phospho-Tuberin/TSC2 (Thr1462) (#3617), Phospho-LCK (Y394) [Phospho-Src Family (Tyr416), #6943], Phospho-ZAP-70 (Tyr319)/SYK (Tyr352) (#2717), CBL-B (#9498), GAPDH (#2118), AKT (#2920S), phosphorylated AKT T308 (#4056), phosphorylated AKT Ser473 (#4058), phosphorylated LAT Tyr191 (#3584), LAT (#45533), phosphorylated SHIP1 Tyr1020 (#3941), p-SHIP2 Y986 Y987 (#2008S), SHIP1 (#2728), phosphorylated PI3 Kinase p85 (Tyr458)/p55 (Tyr199) (#17366), PI3K-85 (#4257S), phosphorylated ERK Phospho-p44/42 MAPK (ERK1/2, Thr202/Tyr204, #4370), PTEN (#9188), and pS6 (Ser 235/236, #4858). The following day, the blots were rinsed and incubated with HRP-conjugated secondary antibodies. The blots were detected with a chemiluminescent substrate and a Bio-Rad Chemi-Doc Imaging system.

### Luciferase Assay of 3′UTR Activities of IL-2 and IFN-γ mRNA.

Murine 3′UTR of IL-2 mRNA or IFN-γ mRNA was cloned into the dual-luciferase vector (psicheck2) (Promega, C8021) using XhoI and NotI sites. The Psicheck2 plasmid containing 3′UTR of IL-2 or IFN-γ mRNA was transduced into hM3Dq/β1 T cells prestimulated with TCR (1 μg/mL anti-CD3 + 2 μg/mL anti-CD28) + IL-2 (40 U/mL) by Neon transfection system (Invitrogen MPK5000 and Neon 100 μL kit cat MPK10096) following the manufacturer’s instruction. Cells were harvested, washed, and resuspended in buffer T at 4 × 10^7^ cells/mL. The Psicheck2 plasmid was added at 100 μg/mL. Electroporation settings were 1,550 V, 10 ms intervals, three pulses. T cells were cultured with IL-2 (40 U/mL) for 1 to 2 d after transduction. Luciferase assays were performed by using dual-luciferase reporter assay system (Promega, E1910) and TriStar^2^ LB942 multimode reader (Berthold Technologies). In brief, 5 × 10^5^ cells/sample were cultured with 10 μM clozapine ± anti-1 μg/mL anti-CD3 ± 2 μg/mL anti-CD28 for 5 h. The cells were washed with PBS and then lysed in 50 μL passive lysis buffer (Promega, E194A). One hundred microliters of luciferase assay buffer (LSRII) was added to 20 μL of cell lysate. The plates were read by TriStar^2^ LB942 multimode reader with a setting of 10 s. One hundred microliters of Stop and Glo buffer was added to the sample. The plates were read again at the same setting. To measure the activities of 3′UTR, the Renilla (the Stop and Glo readings) was divided by firefly reading (the LSRII reading).

#### qRT-PCR.

Total RNA from naïve and memory CD25^−^Va2^+^CD4^+^ cells were isolated with RNeasy (Qiagen). Complementary DNA was synthesized using a cDNA synthesis kit (Quantabio) according to the manufacturer’s instruction. Expression of *Il-2, Ifng, Il-4, or Il-13* mRNA was measured using a commercial primer/probe set (Life Technologies, Mm00434256_m1 *Il2*; Mm01168134_m1 *Ifng*, Mm00445259_m1 *Il4*, Mm00434204_m1 *Il13*). Quantitative PCR was performed using a 7900HT Fas Real-Time PCR system (Applied Biosystems) with the following cycles: 50 °C for 2 min, 95 °C for 10 min, 40 cycles of 95 °C for 15 s, and 60 °C for 1 min. Relative expression was normalized to *β2μ *(IDT, Mm.PT.39a.22214835).

### Statistical Analysis.

All data were displayed as mean ± SD. Statistical analysis was done using an unpaired two-tailed Student’s *t* test. A *P* value < 0.05 was considered significant.

## Supplementary Material

Appendix 01 (PDF)

## Data Availability

All study data are included in the article and/or *SI Appendix*.
