# Supplementary material for: Stimulation of ectopically expressed muscarinic receptors induces IFN-γ but suppresses IL-2 production by inhibiting activation of pAKT pathways in primary T cells
Source: Proc Natl Acad Sci U S A. 2023 Jun 12;120(25):e2300987120. doi: 10.1073/pnas.2300987120 (PMC10288620; doi:10.1073/pnas.2300987120)
Supplement: Supplementary file 1 — Appendix 01 (PDF) [file pnas.2300987120.sapp.pdf]

Supplementary Information for

Stimulation of ectopically expressed muscarinic receptors induces IFN-γ but suppresses IL-2 production by inhibiting activation of pAKT pathways in primary T cells

Trang T. T. Nguyen[1], Wen Lu[1], Wandi S. Zhu[2], K Mark Ansel[2], Hong-Erh Liang[1], and Arthur Weiss[1]

[1]Division of Rheumatology, Department of Medicine, University of California, San Francisco, San Francisco, CA, USA. [2]Department of Microbiology and Immunology, University of California San Francisco, San Francisco, CA 94143, USA.

Correspondence: arthur.weiss@ucsf.edu

Running Title: Activating T cells with ectopically expressed muscarinic receptors

This PDF file includes:
Supplementary text
Figs. S1, S2 and S3

Fig. S1

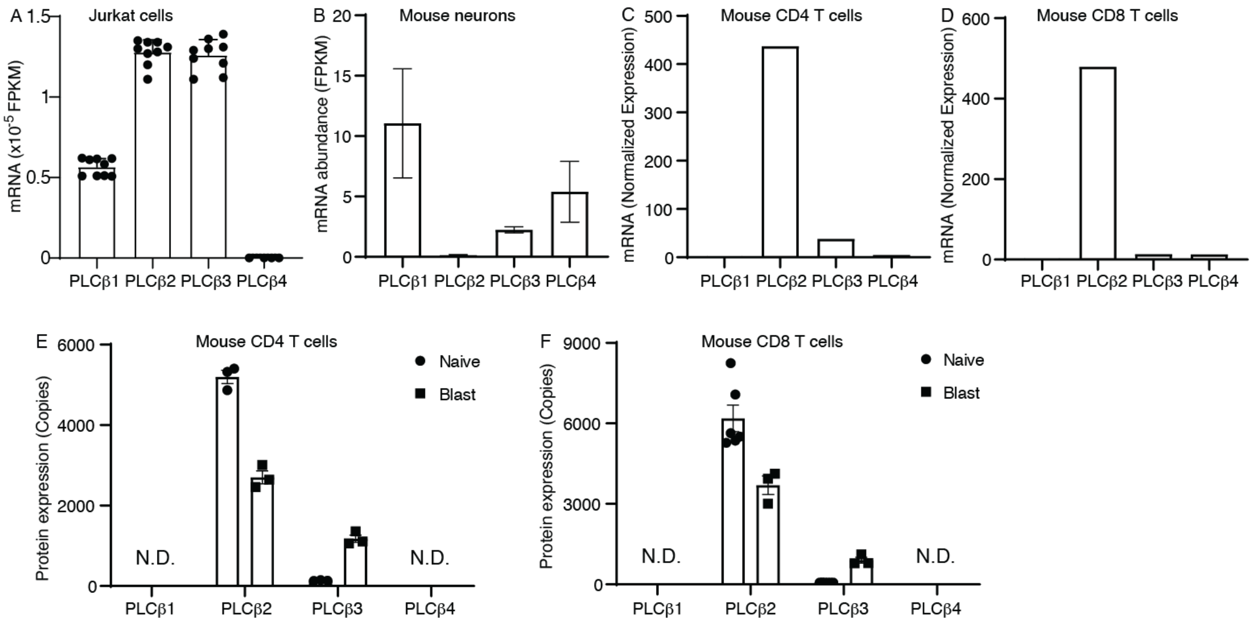

Fig. S1. While Jurkat cells and mouse neurons express PLCβ1, mouse T cells do not express PLCβ1. (*A*) mRNA expression of different PLC isoforms in Jurkat cells (1) (GEO: GSE45428). (*B*) mRNA expression of PLC isoforms in mouse neurons (from brainrnaseq.org). (*C*-*D*) mRNA expression of PLC isoforms in mouse CD4 and CD8 T cells (from Immgen.org). (*E*-*F*) Protein expressions of PLC isoforms in mouse CD4 and CD8 T cells (2). N.D., not detected.

Fig. S2

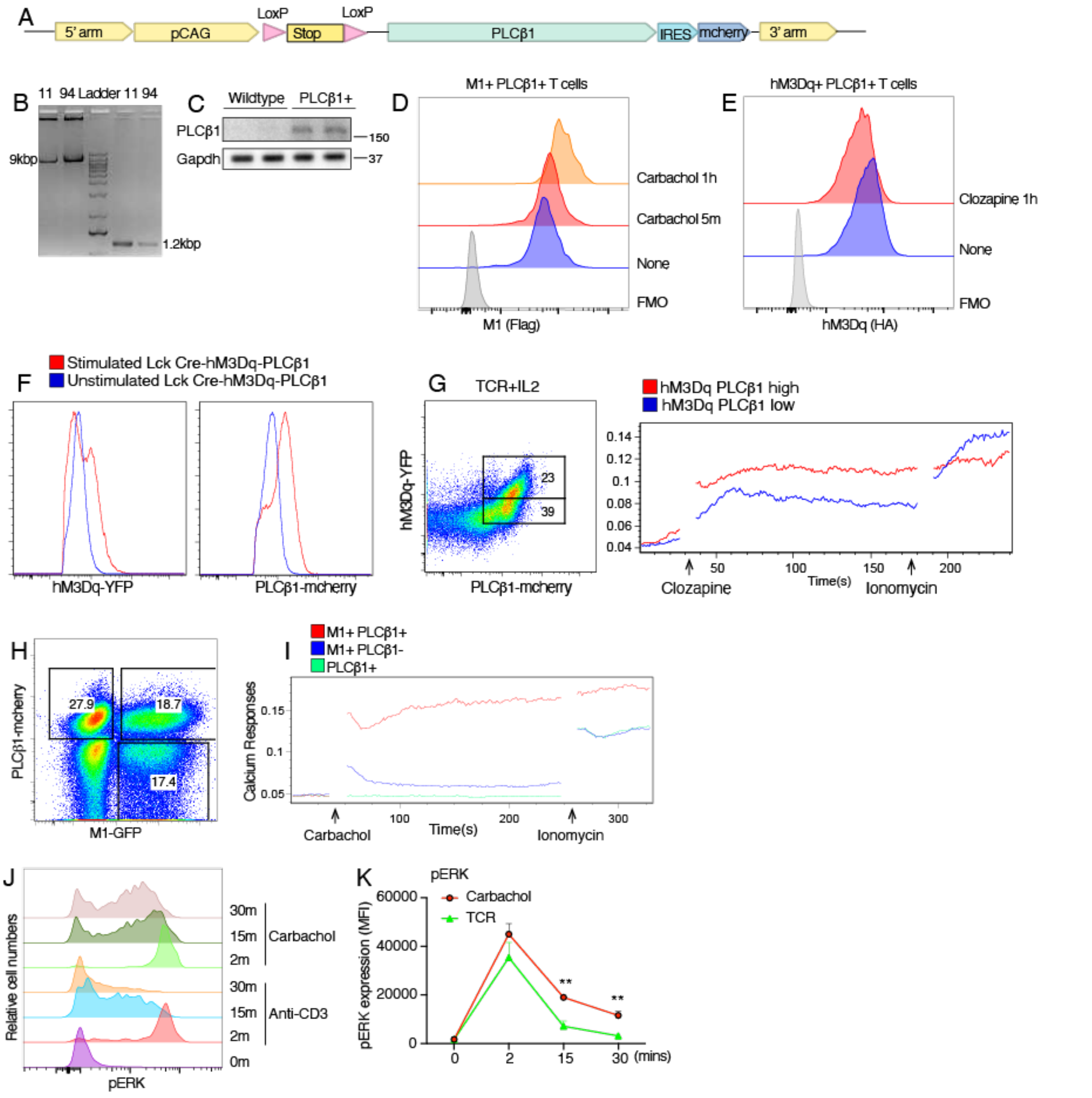

Fig. S2. PLCβ1 greatly enhances calcium responses in activated muscarinic receptors in primary T cells. (*A*) Map of vector containing PLCβ1 targeting Rosa26 locus to generate inducible Plcβ1 knock-in mice. (*B*) PCR left and right Rosa26 arm of genomic DNA isolated from 2 representative ES clones (11 and 94) containing Plcβ1-IRES-mcherry knocked in Rosa26. Genomic DNA from each ES colony was screened by 5' arm PCR using primers covering Rosa26 sequence outside the 5′ homologous arm and the pCAG gene; and 3' arm PCR using primers covering Rosa26 sequence outside the 3′ homologous arm and mcherry gene (3' forward and reverse primers: ACACCATCGTGGAACAGTACGAACG, and CAGCTTGCCTTCGGGAAATGAGCCG; 5' forward and reverse primers:

AGAGCCTCGGCTAGGTAGGGGATCG and CATCATCAAGGAAACCCTGGACTAC). Expected sizes of PCR products for 3' and 5' PCR are 9 and 1.2kb, respectively.
(*C*) Immunoblot analysis of Plcβ1 in wildtype and Lck-Cre x hM3Dq x Plcβ1 CD4 T cells. (*D*) Flow cytometry of M1+ Plcβ1+ CD4 T cells stained for Flag staining (M1) over time after carbachol stimulation. (*E*) Flow cytometry of Lck-Cre x hM3Dq x Plcβ1 CD4 T cells stained for HA staining (hM3Dq) over time after clozapine stimulation. (*F*) FACS plots showed hM3Dq-YFP and Plcβ1-mcherry expression in unstimulated or stimulated (anti-CD3 + anti-CD28 + IL-2) primary Plcβ1+ CD4 T cells transduced. (*G*) Calcium changes in hM3Dq Plcβ1 high or hM3Dq Plcβ1 low CD4 T cells in response to clozapine (hM3Dq agonist, 10μM) and ionomycin (1μM) over time. (*H*) FACS plots showed primary Plcβ1+ CD4 T cells transduced by retrovirus transfer vectors encoding M1-GFP. (*I*) Calcium changes in transduced primary CD4 T cells in response to carbachol (M1 agonist, 500μM) and ionomycin (1μM) over time. (*J*) Overlaid histograms and (*K*) bar chart showing mean fluorescent intensity (MFI) of phosphorylated Erk in M1+ Plcβ1+ CD4 T cells in response to 500μM carbachol or 10μg/ml anti-CD3 followed by cross-linking with 20μg/ml anti-Armenian hamster IgG over time. P *< 0.05, **<0.005, ***<0.0005.

Fig. S3

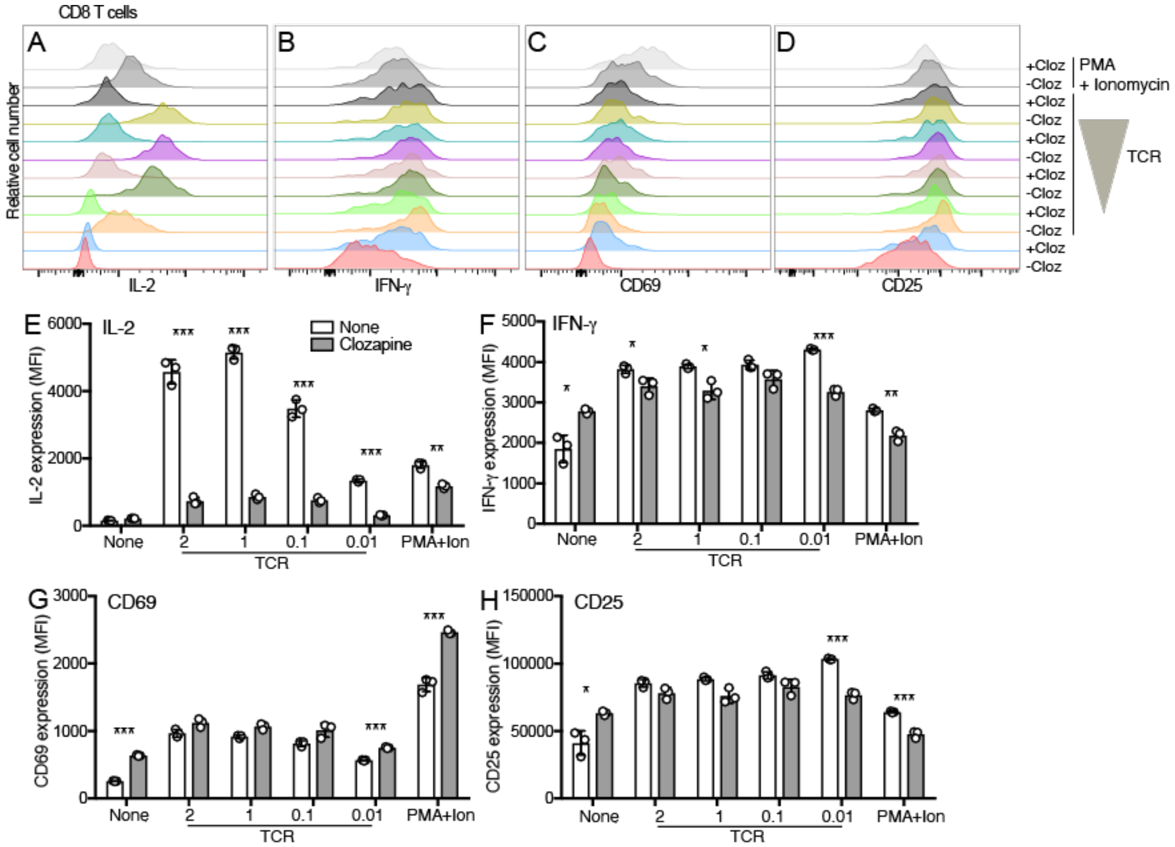

Fig. S3. Stimulation of muscarinic receptors induces IFN-γ, CD69, CD25 but not IL-2 in hM3Dq+Plcβ1+ CD8 T cells. CD8 T cells from LckCre-hM3Dq-Plcβ1 mice were activated with anti-CD3 (1μg/ml) + anti-CD28 (2μg/ml) and IL-2 (40U/ml) for 3 days and then cultured with IL-2 (40U/mL) for 2 days. (*A-H*) hM3Dq/β1 CD8 T cells in response to 10μM clozapine (cloz) and/or TCR stimulation (2, 1, 0.1, or 0.01 μg/ml anti-CD3 + 2μg/ml anti-CD28) and/or PMA (50ng/ml) + ionomycin (ion, 1μM) for 16 hours. (*A-D*) Overlaid histograms of IL-2, IFN-γ, CD69, CD25 expression in hM3Dq/β1 CD8 T cells. (*E-H*) Bar chart shows MFI of IL-2, IFN-γ, CD69, CD25 expression in hM3Dq/β1 CD8 T cells. Data are representative of three independent experiments. P *< 0.05, **<0.005, ***<0.0005.

References

1. G. M. Sheynkman, M. R. Shortreed, B. L. Frey, L. M. Smith, Discovery and Mass Spectrometric Analysis of Novel Splice-junction Peptides Using RNA-Seq. *Molecular & Cellular Proteomics* **12**, 2341–2353 (2013).

2. A. J. M. Howden, *et al.*, Quantitative analysis of T cell proteomes and environmental sensors during T cell differentiation. *Nat Immunol* **20**, 1542–1554 (2019).
